# Role of saturated fatty acid metabolism in posttranslational modifications of the Tau protein

**DOI:** 10.1007/s11010-025-05275-2

**Published:** 2025-04-10

**Authors:** Valeria Melissa García-Cruz, Roberto Coria, Clorinda Arias

**Affiliations:** 1 Departamento de Medicina Genómica y Toxicología Ambiental, Instituto de Investigaciones Biomédicas, Universidad Nacional Autónoma de México, 04510 Mexico City, Mexico; 2 Departamento de Bioquímica y Biología Estructural, Instituto de Fisiología Celular, Universidad Nacional Autónoma de México, 04510 Mexico City, Mexico

**Keywords:** Tau, Saturated fatty acids, Protein kinases, Deacetylases, Energy metabolism

## Abstract

The relationship between metabolic alterations induced by the consumption of a high-fat diet (HFD) and the risk of developing neurodegenerative diseases such as Alzheimer’s disease (AD) has been extensively studied. In particular, the induction of neuronal insulin resistance, endoplasmic reticulum stress, and the production of reactive oxygen species by chronic exposure to high concentrations of saturated fatty acids (sFAs), such as palmitic acid (PA), have been proposed as the cellular and molecular mechanisms underlying cognitive decline. Lipid metabolism affects many processes critical for cellular homeostasis. However, questions remain as to whether neuronal exposure to high sFA levels contributes to the onset and progression of AD features, and how their metabolism plays a role in this process. Therefore, the aim of this work is to review the accumulated evidence for the potential mechanisms by which the neuronal metabolism of sFAs affects signaling pathways that may induce biochemical changes in the AD hallmark protein Tau, ultimately promoting its aggregation and the subsequent generation of neurofibrillary tangles. In particular, the data presented here provide evidence that PA-dependent metabolic stress results in an imbalance in the activities of protein kinases and deacetylases that potentially contribute to the post-translational modifications (PTMs) of Tau.

## Introduction

Lifestyle factors, including the consumption of unhealthy diets high in energy density, are associated with pathological brain aging. Chronic intake of a high-fat diet (HFD) rich in saturated fat contributes to metabolic diseases such as obesity, insulin resistance, metabolic syndrome, and type 2 diabetes, which promote brain aging and increase the risks of cognitive decline and even Alzheimer´s disease (AD) [1–4]. In fact, researchers have proposed that intervention strategies designed to modify some of these metabolic risk factors could prevent 14% of dementia cases [5]. Several studies have provided convincing data on the central role of lipids in AD, as evidenced by their potential dysfunction in the maintenance of membrane integrity and remodeling, plasticity mechanisms, energy balance, and neuroinflammation [6–10]. However, at present, it has not clearly determined which lipid metabolism routes are directly connected to neuronal changes that may lead to the expression of specific biochemical and histopathological markers of AD, such as amyloid production and Tau phosphorylation.

Long-chain saturated fatty acids (sFAs) are the main component of an HFD, and their high consumption impacts the brain lipid composition and has been suggested to be key players in the development of obesity, insulin resistance, and systemic inflammation [11–13]. The involvement of sFA metabolism in Tau protein PTMs similar to that observed in AD brains has also been proposed [14–16].

Due to the significant role of lipids in the pathophysiology of AD, in this review, we summarize some of the evidence regarding the major mechanisms by which exposure to high levels of sFAs can lead to neuronal dysfunction and the signaling pathways involved. We have also reviewed the existence of a link between sFA metabolism and the biochemical changes underlying some Tau PTMs.

## Post-translational modifications of Tau

Tau is responsible for axonal microtubule stability, allowing axonal transport and the establishment of neuronal polarity for proper neuronal function. In humans, Tau is encoded by a single gene located at chromosome 17 that is transcribed in an RNA that yields, by alternative splicing, six isoforms [17]. Following biochemical modifications, the Tau protein detaches from microtubules and can produce insoluble aggregates implicated in the formation of neurofibrillary tangles (NFTs), a hallmark of AD. Tau undergoes several PTMs, with phosphorylation being the most important because of the large number of serine, threonine, and tyrosine residues present throughout its primary structure. Other frequent PTMs, such as acetylation and ubiquitination, also play roles in regulating Tau physiology and pathology. Tau is also subjected to methylation, glycosylation, SUMOylation, nitration, and oxidation, which are present less frequently [18].

## Phosphorylation

As mentioned above, phosphorylation is the most frequent PTM throughout the Tau protein, which contains 85 putative sites for the addition of phosphate groups. Physiologically, the degree of phosphorylation in its microtubule-binding domain and C-terminal region determines the extent of Tau binding to microtubules, thereby regulating microtubule stability. Normally, 3–5 mol of phosphate are present per Tau monomer; however, hyperphosphorylation at some specific residues, such as S214, T231, S235, S262, S293, S324, and S356, decreases the affinity of Tau for microtubules. Thus, the addition of phosphate groups adds a negative electric charge to Tau that can disrupt its molecular interactions, negatively affecting axonal stability and transport, with detrimental consequences for neurons [18]. The phosphorylation rate of this protein is primarily driven by the balance between protein kinase and phosphatase activities. Some of the main kinases involved in Tau phosphorylation include proline-directed kinases, such as GSK3β, PKA, CK1, CK2, MAPKs, and CDK5, as well as non-proline-directed kinases, including AMP-activated kinases like MARK4; Ca^2+^-dependent kinases like CAMK2; and tyrosine-directed kinases of the Src family, such as Fyn [19]. Interestingly, GSK3β, which is central to AD pathology, preferentially phosphorylates Tau, which is primed by other kinases [19, 20]. On the other hand, the protein phosphatase PP2A, which is the most abundant phosphatase in the brain, is responsible for more than 70% of Tau dephosphorylation [21]. Specific inhibition of PP2A is sufficient to induce Tau hyperphosphorylation and neurodegeneration in primary cortical neurons [22].

## Acetylation

Acetylation is the addition of an acetyl group to lysine residues; the function of this modification on Tau has been associated to the regulation of phosphorylation levels. Specifically, acetylation of the KXGS motifs, which are present in the microtubule-binding region of the protein, inhibits the phosphorylation of these sites, preventing protein aggregation [23]. Structural studies have shown that acetylation can promote the interaction of Tau monomers, leading to the formation of helical or straight filaments, depending on the location of this PTM [24]. In fact, elevated Tau acetylation levels have been reported as early event preceding the formation of NFTs [25]. Acetylation at specific residues inhibits Tau degradation by chaperone-mediated autophagy, promoting the release of Tau-containing vesicles into the extracellular space and potentially contributing to the possible spread of Tau-associated pathology [26]. However, contrasting reports suggest that acetylation of residues K274, K290, K231, and K353 promotes Tau interaction with chaperone proteins inducing Tau degradation [27].

## Ubiquitination

The addition of ubiquitin (Ub) to lysine residues is an ATP-dependent process that requires the action of the enzymes E1, E2, and E3 ubiquitin ligases, which tag Tau for degradation by the ubiquitin–proteasome system. Seventeen putative ubiquitination sites on lysine residues in pathological Tau, mainly located in the microtubule-binding domain, have been identified [28]. The interplay between phosphorylation, acetylation, and ubiquitination appears to modulate Tau solubility and turnover, with phosphorylation being a cooperative modulator [29], whereas lysine acetylation serves as a negative regulator [25]. Experimental evidence indicates that a certain level of protein phosphorylation is necessary for recognition and ubiquitination by E3 ligase enzymes, such as CHIP-Hsc70, a mechanism that contributes to the degradation of p-Tau via the proteasomal pathway [30].

## Tau modifications in Alzheimer's disease

Between 43 and 55 different phosphorylation sites, 19 acetylation sites, 14 to 17 ubiquitination sites, and 4 methylation sites have been identified in the Tau protein from AD brains [31]. Proteomic studies have revealed positive correlations between the PTMs of Tau and disease progression, which aligns with the Braak stages [32]. During normal brain aging, only few phosphorylated sites are found, corresponding mainly to residues T181, T231, S235, S400, T403, and S404. In the early stages of AD, the most frequently phosphorylated sites are S199/202, T212/217, S237, S262, and S396. At later stages, in samples from patients with Braak stages 3 and 4, the number of phosphorylated residues increases considerably. Additionally, other PTMs, such as ubiquitination and acetylation, begin to occur, with residues K274, K311, and K369 being the most frequently detected. Finally, in samples from patients at advanced stages of the disease (corresponding to Braak stages 5–6), the presence of PTMs is substantially increased [31]. At this advanced AD stage, the Tau protein can interact with components of the nuclear pores, disturbing nucleocytoplasmic transport, and contributing to aggravated neurotoxicity [33]. Augustinack and colleagues reported the differential presence of Tau phosphorylation at specific residues in various stages of tangle formation: pretangles (T231, S262, and T153), intraneuronal tangles (T175/181, S262, S356, S422, S46 and S214) and extraneuronal tangles (S199, S202, S214, S396, S404, T205, and T212) [34]. Similarly, Kimura and colleagues identified phosphorylation at S199, S202 and S409 in the pre-aggregation stage and at S396 and T231 in already formed tangles [35], suggesting a possible sequence of biochemical events involved in Tau aggregation, though early cellular events remain unknown. Ubiquitin tagging on Tau has been detected in both the early and intermediate stages of the disease; however, Ub tagging at the N-terminus of the protein is specifically associated with early conformational changes in the paired helical filament (PHF) conformation [36]. In this same PHF-type conformation, Ub chains on K48, K11 and K6 have been observed [37]. In brain homogenates from the AD patients, both monoubiquitination and polyubiquitination coexist, with the latter predominantly found in the microtubule-binding region, specifically at residues K254, K311, and K353. These modifications may serve as markers of resistance to Tau degradation [37]. Tau acetylation at K274 and K281 has been correlated with memory problems, AMPA-type receptor trafficking, and the blockade of postsynaptic activity linked to AD [38]. The acetylation of these same residues causes a cellular mislocalization of Tau, displacing it from neuronal axons to the somatodendritic compartment and compromising cytoskeletal stability and functionality, and subsequent axonal transport [39]. Acetylation at K280 is predominant in the brains of patients with advanced stages of the disease but is not observed in the brains of healthy subjects [31, 40–42].

Despite increasing knowledge regarding the mechanisms underlying Tau PTMs that ultimately promote its aggregation into NFTs, the nature of a precipitating factor remains elusive. Given the imbalance between the activities of protein kinases/phosphatases and acetylases/deacetylases, as well as the deregulation of the ubiquitin–proteasome pathway and autophagy systems, energy metabolic changes and abnormal lipid metabolism can be suggested as contributing factors to Tau PTMs.

## Neuronal metabolism of saturated fatty acids

Saturated fatty acids (sFAs) are essential for brain development, and in adults, they serve not only as building blocks for nearly all types of structural lipids but also as precursors for bioactive lipids and for the fatty acylation of proteins. sFAs can be categorized according to their chain length into short-chain (2–4 carbons), medium-chain (6–12 carbons), long-chain (14–20 carbons), and very long-chain (22 or more carbons). Long-chain saturated fatty acids account for 80–90% of the total sFAs derived from dietary intake, with palmitic acid (PA) being the most abundant. FAs travel through the systemic circulation and reach the central nervous system by crossing the blood–brain barrier (BBB) via different transport systems. They can cross by passive transport or utilize various transport systems, including fatty acid translocase/CD36, fatty acid transporter proteins (FATPs), and type 1 caveolins. Additionally, sFAs can be delivered by the hydrolysis of lipoproteins at the BBB [43–46]. Similar to the BBB, neuronal uptake of sFAs is specific and depends on chain length, primarily involving FATPs. In general, the concentration of sFAs in the brain is proportional to that found in the circulation [47]; therefore, metabolic conditions that elevate plasma sFA levels influence its accumulation in the brain. Obese individuals with metabolic syndrome show a 50% greater brain uptake of sFAs [48], suggesting that under these metabolic conditions, neurons are exposed to higher concentrations of these lipids. Additionally, an increase in FATP1 transporter levels in the prefrontal cortex of obese rats has also been reported [49], providing further evidence of the impact of systemic sFA circulation on brain lipid levels and metabolism. Compared with healthy individuals, patients with type 2 diabetes exhibit a 1.3- to 3.5-fold increase in plasma PA concentration [50]. In this context, a negative correlation has been observed between elevated PA concentrations in the cerebrospinal fluid of obese individuals and memory performance [51]. Furthermore, increased PA concentrations have been detected in the parietal cortex of AD patients [52].

Within the brain, fatty acid-binding proteins can capture sFAs and transport them to different cellular compartments to be metabolized via several pathways [45]. Under normal conditions, astrocytes are the primarily brain cells capable of utilizing sFAs for generate ATP through mitochondrial β-oxidation, whereas neuronal cells have limited capacity for FA-dependent energy production [53, 54]. An exception to this paradigm is specialized hypothalamic neurons, which can sense and metabolize circulating FAs through β-oxidation to maintain whole-body energy homeostasis [47, 55, 56]. The reduced capacity of neurons for mitochondrial β-oxidation is attributed to the limited transport of long-chain FAs across the mitochondrial membrane [54]. However, neurons do possess the enzymes carnitine palmitoyltransferase 1 (CPT1), which is responsible for changing CoA to carnitine, and carnitine palmitoyltransferase 2 (CPT2), which replaces carnitine with CoA inside the mitochondria and releases acyl-CoA for β-oxidation [57]. CPT1 has low activity but is present at high concentrations in neuronal mitochondria [58, 59], and interestingly, CPT2 deficiency results in brain deficits [60, 61], indicating that fatty acyl-CoA transport inside mitochondria plays a key role in proper brain function. The low rate of sFA oxidation in the brain under normal conditions has also been attributed to the high content and high activity of thioesterase 7 (ACOT7), an enzyme responsible for hydrolyzing long-chain fatty acyl-CoA that may disturb the flux of long-chain FAs through various metabolic pathways [54, 62]. Interestingly, deficiency of this thioesterase is associated with neurological deficits and even with neurodegeneration [62]. Another enzyme that is downregulated in neurons is 3-ketoacyl-CoA thiolase, which catalyzes the final three steps of long-chain sFA β-oxidation. Its activity in neuronal mitochondria is 125-fold lower than in heart mitochondria [63]. Although sFA β-oxidation is relatively low in adult neurons, this metabolic pathway may be activated under specific conditions. When neurons are exposed to high concentrations of sFAs, they can use them as metabolic fuel, resulting in metabolic disadvantages. In particular, β-oxidation demands more oxygen consumption and generates superoxide, with negative consequences for neuronal homeostasis [54]. In fact, the increased generation of reactive oxygen species is considered a key factor in brain aging and neurodegenerative diseases [64]. The oxidation of palmitoyl carnitine has been observed in neuronal mitochondria in the presence of various metabolic substrates, as well as under conditions of high energy demand. This has been demonstrated in retinal photoreceptors, which metabolize PA through the oxidative pathway [65, 66]. Moreover, in differentiated neuroblastoma cells, PA energy metabolism has been implicated in the development of insulin resistance associated with ATP production through mitochondrial metabolism [67]. Interestingly, when cultured hippocampal neurons are exposed to high concentrations of PA, the expression levels of proteins involved in FA metabolism and inflammatory mediators increase [68]. Although a small proportion of β-oxidation may occur in neuronal peroxisomes, only very long-chain FAs are partially degraded into short-chain FAs, which are then completely oxidized in mitochondria [69]. Notably, in patients with early-stage AD, peroxisomal dysfunction is characterized by reduced levels of these organelles in neurites containing phosphorylated Tau in pretangles [70].

Astrocytes have the capacity to metabolize sFAs via different metabolic pathways. Despite their ability to produce energy through β-oxidation, in the presence of high contents of sFAs, they may produce neurotoxic levels of ceramides that have been implicated in neurodegeneration [71]. Conversely, astrocytes play a protective role by preventing neuronal lipotoxicity, activating mitochondrial oxidative phosphorylation, and inhibiting of lipid droplet accumulation in neurons [72]. However, lipid-laden reactive astrocytes stimulate sFA oxidation in neurons, leading oxidative stress in these cells [72]. These findings suggest that the loss of astrocyte-dependent oxidative metabolism upon sFAs exposure triggers a maladaptive metabolic response in neurons, characterized by FA oxidation, ROS production, and neurodegeneration.

In addition to serving as energy source, PA also follows several physiological routes in neurons. These include lipid synthesis for membrane remodeling during brain plasticity events [73]; protein modification via palmitoylation, which regulates protein stability, trafficking, endocytosis, and recycling [74, 75]; and the production of ceramides, which are bioactive molecules that are considered key mediators of neuronal development, death, and senescence [76, 77].

## Saturated fatty acids and Alzheimer’s disease

Metabolic risk factors for AD include diabetes, midlife obesity, increased blood pressure, and physical inactivity [78]. Abnormal lipid metabolism is largely considered a key factor contributing to pathological brain aging and neurodegeneration. Notably, lipid droplets are present in various brain cells, including neurons, throughout aging and at early stages of AD, suggesting the activation of a cellular response to mitigate the toxic effects of exposure to sFAs [79]. These lipids can access both neurons and glial cells from high systemic circulating levels [80]. Interestingly, genome-wide association studies have identified the presence of polymorphisms in several lipid transport and metabolism genes in patients with sporadic AD [81, 82]. In a Swedish twin study, it was observed that overweight and obesity in midlife increased the risk of dementia in later life, regardless of the presence of diabetes or vascular disorders, suggesting that both environmental and genetic factors are linked to altered metabolic lipid handling [83]. More recently, an epidemiological study revealed that saturated long-chain FAs present in the circulation predict the risk of AD among participants with mild cognitive impairment [84]. Several studies using experimental models have also revealed the relationship between exposure to high lipid concentrations and the expression of AD hallmarks. Memory deterioration accompanied by a reduction in autophagy, synaptic dysfunction, and increased levels of Tau phosphorylation were observed in C57BL/6 mice fed an HFD for 22 weeks [85]. An HFD aggravated amyloid and Tau pathology in 3xTg-AD mice [86], highlighting the role of saturated fat exposure in declining cognitive performance and potentially in the PTMs of the Tau protein. Recently, a strong association was reported between aging and elevated serum levels of palmitoyl carnitine, implicated in the induction Tau phosphorylation and mitochondrial dysfunction in neuronal cells [87].

**Fig. 1:**
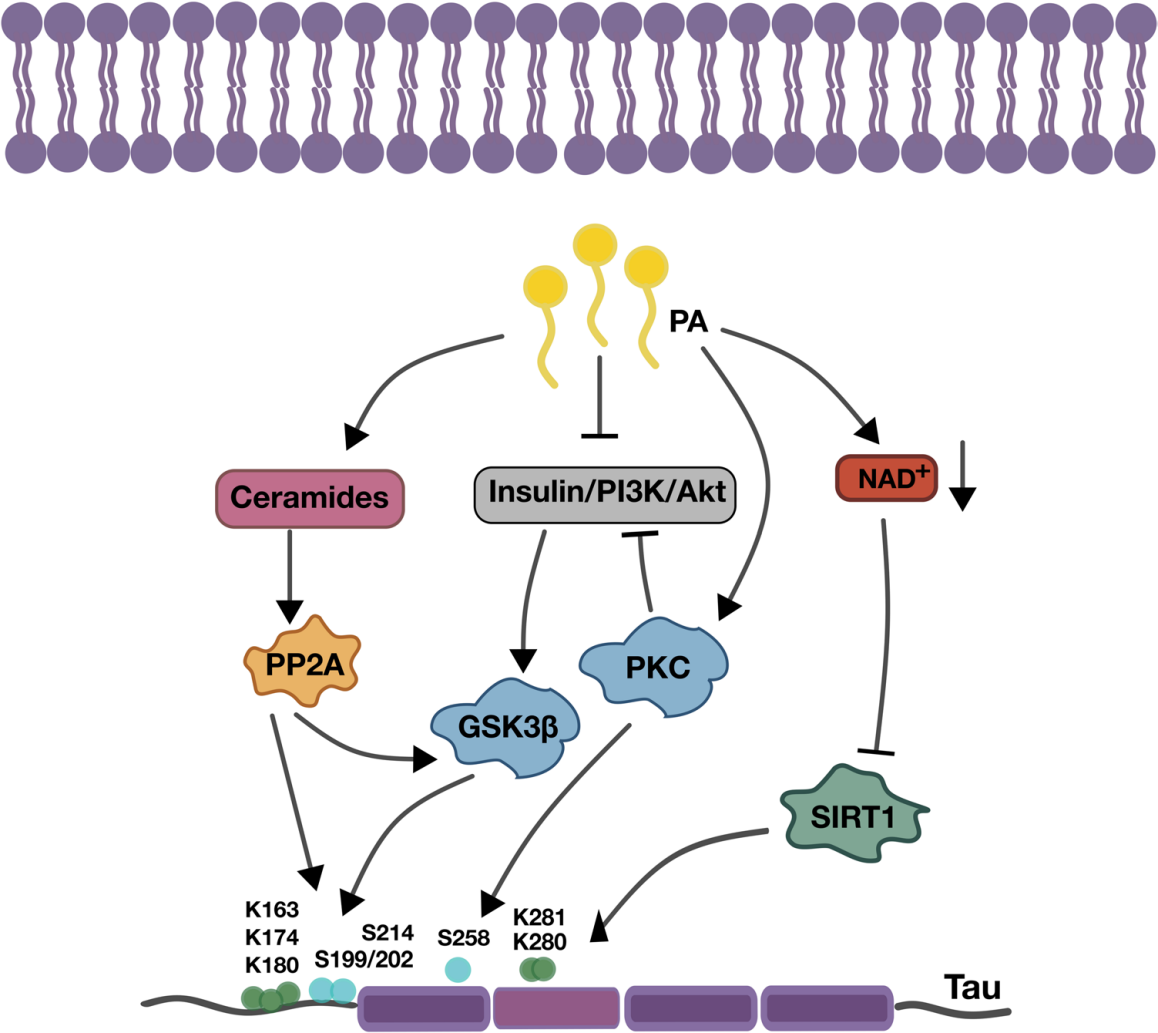
Effects of neuronal metabolism of sFA that alter the activities of protein kinases, phosphatases and desacetylases. High PA metabolism in neurons can lead to an increased ceramide levels, reduced insulin/PI3K/Akt pathway and NAD^+^ levels, affecting Tau phosphorylation and acetylation at specific residues

Despite the growing knowledge that exposure to high levels of sFAs may be linked to neuronal damage through several biological pathways, the precise mechanisms underlying these associations, particularly those impacting the expression of AD-related hallmarks, appear to be multifactorial and complex. Some of the plausible signaling pathways altered by PA metabolism are discussed in the next section Fig. 1.

## PA metabolism and cellular signaling pathways that may impact Tau phosphorylation

Although the circumstances under which Tau becomes modified and accumulates as insoluble species are currently unknown, one line of evidence supports the idea that an imbalance in neuronal metabolic routes may contribute to Tau changes. Many studies have addressed the effects of sFA metabolism on different cellular signaling pathways important for brain homeostasis. After only 3 days of HFD consumption, different cellular pathways involved in metabolism (28%), signaling (2%), and cellular stress (12%) are affected [88]. One signaling pathway directly altered by PA in neurons is the insulin/PI3K/Akt pathway [67, 89–91]. While information regarding the effects of PA on brain insulin resistance has been provided mostly from studies of hypothalamic neurons [92, 93], the analyses of other neuronal cell models have also shown that insulin signaling is blunted by PA. Insulin resistance provokes increases in β-secretase activity, Aβ_1–40/42_ production, and Tau hyperphosphorylation through the insulin-dependent activation of the atypical kinase PKC-λ/ι in mice and monkeys [94]. Moreover, PA exposure increases the nuclear p-NF$k$B levels and reduces the insulin-dependent activation of Akt in neuroblastoma cells [95]. Since Akt activation phosphorylates GSK3β at the inhibitory S9 residue, the PA-induced reduction in Akt activates GSK3β, which has been implicated in Tau phosphorylation under these circumstances [16, 96]. GSK3β has been identified as one of the major enzymes mediating the hyperphosphorylation of at least 23 Tau residues involved in tauopathies, including AD [97–100]. Chronic neuronal exposure to PA may thus impact the phosphorylation/dephosphorylation balance in neurons, leading to the hyperphosphorylation of the Tau protein. Inhibition of the insulin pathway, which reduces Akt activity, can also decrease the expression of the chaperone CHIP, an ubiquitin ligase that responds to cellular stress and regulates the degradation of Tau through the HSP90/CHIP complex [101]. In cortical neurons and differentiated human neuroblastoma cells, the PA-dependent reduction in insulin signaling activates the nutrient-sensing kinase mTOR complex C1 and increases intracellular Ca^2+^ levels, which in turn, activate protein kinase C⍺ (PKC⍺) [16, 67, 90]. Recently, it was reported that PA profoundly increases the production of acetyl-CoA from β-oxidation, which triggers mTORC1 activity in a croaker model [102]. Although mTOR is not directly implicated in Tau phosphorylation, its activation indirectly reduces insulin signaling, leading to the activation of GSK3β and, subsequently, activating S6K, a kinase that regulates Tau phosphorylation [103, 104]. In fact, S6K was found to be activated in NFTs in brain samples from AD patients [103]. In the case of classical PKC⍺, only the phosphorylation of Tau at S258 is relevant for AD [100].

Changes in intraneuronal Ca^2+^ concentration have been documented as early events involved in the development of certain neuropathological features of AD [105–107]. Evidence has shown that PA metabolism is associated with elevated levels of cytosolic free Ca^2+^ in hypothalamic neurons and differentiated neuroblastoma cells [67, 108] One protein that senses Ca^2+^ levels is calmodulin (CaM), whose interaction with the kinase CaMKII, due to the disruption of Ca^2+^ homeostasis, has been implicated in AD. In fact, multiple CaM binding proteins are critical for learning and memory, as well as for the formation of amyloid plaques and tangles [106]. CaMKII is a complex kinase that participates in synaptic plasticity and potentially regulates the extent of Tau phosphorylation at least at 5 relevant sites [109–111]. Many other pathways in which PA metabolism has been implicated can affect the Tau phosphorylation/dephosphorylation rate. One such pathway involves c-Jun N-terminal kinase (JNK), which is able to phosphorylate 12 Tau residues [112, 113] and is activated by PA-induced endoplasmic reticulum stress [114]. Interestingly, among the kinases mentioned above, the sequential activation of PKA, CaMKII and GSK3β leads to a Tau phosphorylation pattern similar to that found in AD patients and in a rat brain slice model [38].

Evidence suggests that aerobic exercise reduces the levels of pS396-Tau and active GSK3β in mouse models of cerebral ischemia and AD [115, 116], and that consuming antioxidants such as resveratrol reduces tau pathology in neurons, via the insulin-PI3K pathway [117]. Moreover, targeting the transcription factor nuclear factor (erythroid-derived 2)-like 2 (NRF2), a master regulator of the antioxidant response, with n3 polyunsaturated fatty acids (n3 PUFA) results in metabolic benefits, including reduced activation of the JNK, supporting a role of nutritional intervention in attenuating Tau PTMs and neurodegeneration [118, 119].

PA is a central molecule in the de novo biosynthetic pathway of ceramides [120]. Increased levels of ceramides have been found in the serum of patients with mild symptoms of AD, indicating that alterations in this lipid pathway occur in the early stages of AD [121, 122]. C2 ceramide is an activator of the protein phosphatase PP2A, which indirectly promotes the activation of GSK3β through the inhibition of the PI3K/Akt pathway [123]. In fact, neuronal exposure to the conditioned media of PA-treated astrocytes causes GSK3β and CDK5 activation and Tau hyperphosphorylation via PA-dependent ceramide synthesis by astrocytes [71]. Interestingly, the GSK3β inhibitor lithium was found to be effective against ceramide-induced apoptosis via the inhibition of PP2A activity, suggesting that drugs targeting different components of the ceramide signaling pathway could be promising therapeutic agents for AD.

The brain expresses very high levels of the free fatty acid receptor GPR40, which, among other effects, is linked to the activation of the ERK1/2 and MAPK signaling pathways in neurons [124, 125]. The role of GPR40 activation by PA has not been analyzed with respect to its impact on Tau phosphorylation, but the receptor-mediated activation of these kinases, which potentially phosphorylate Tau, suggests an interesting avenue for further studies.

The specific sites of Tau that have been reported to be phosphorylated by various protein kinases influenced by PA metabolism are listed in Table 1.

**Table 1:** Summary of major posttranslational modifications of Tau protein and the enzymes potentially affected by sFA metabolism

Enzyme	PTM’s associated	Reported sites	Reference
GSK3β	Phosphorylation	S46, S198, S199, S202, S210, T175, T181, T205, T212, T217, T231, S235, S396, S404, S416, S422	[100, 126, 127]
PKA	Phosphorylation	S199, S202, S210, S214, S235, S262, T205, T212, T217, T231, S356, S409	[100, 127]
PKC	Phosphorylation	S214, S258, S293, S324, S352, S262,	[100, 127]
CAMKII	Phosphorylation	S214, S235, S262, S396, S404, T217, T231, S418, T212, T135	[100, 126, 127]
JNK	Phosphorylation	S199, S202, S235, S396, S404, S422, T181, T205,T212, T217, S404, S422	[100, 127]
p70S6 K	Phosphorylation	S262, S214, T212	[127, 128]
AMPK	Phosphorylation	S262, S396, S404, T231	[107, 127]
PKB/AKT	Phosphorylation	S214, T212	[127]
Abl	Phosphorylation	T394, Y18	[100, 127, 129]
MARK2	Phosphorylation	S262, S356	[101, 130, 131]
Fyn	Phosphorylation	T18	[127, 129]
ERK1/2	Phosphorylation	T69, T153, T175, S356, S396, S404, 422	[100, 132]
mTOR	Phosphorylation	S214, S356, T231	[133]
PP2 A	Dephosphorylation	S199, T205, S214, T231, S235, S262, S356, S404	[100, 127, 134, 135]
PP1 A	Dephosphorylation	T212, T217, S262, S396, S422	[127, 136, 137]
PP3	Dephosphorylation	S199, S202, T205, T212, S214, T217, S235, S262, S396, S404, S409, S422	[127, 136, 138]
P300	Acetylation	K163, K174, K180, K280	[25, 127, 139, 140]
SIRT1	Deacetylation	K163, K174, K180, K280, K281	[25, 127, 139–141]
HDAC6	Deacetylation	KXGS motifs, K280	[23, 127, 142]
Otub1	Deubiquitination	K48 Ubiquitin-Linkages	[40, 131]
CHIP	Ubiquitination	K48 Ubiquitin-Linkages, K257, K259, K267, K274, K281, K290, K321, K343, K353, K375, K385	[131, 143]

## Potential role of PA metabolism in Tau acetylation

**Fig. 2:**
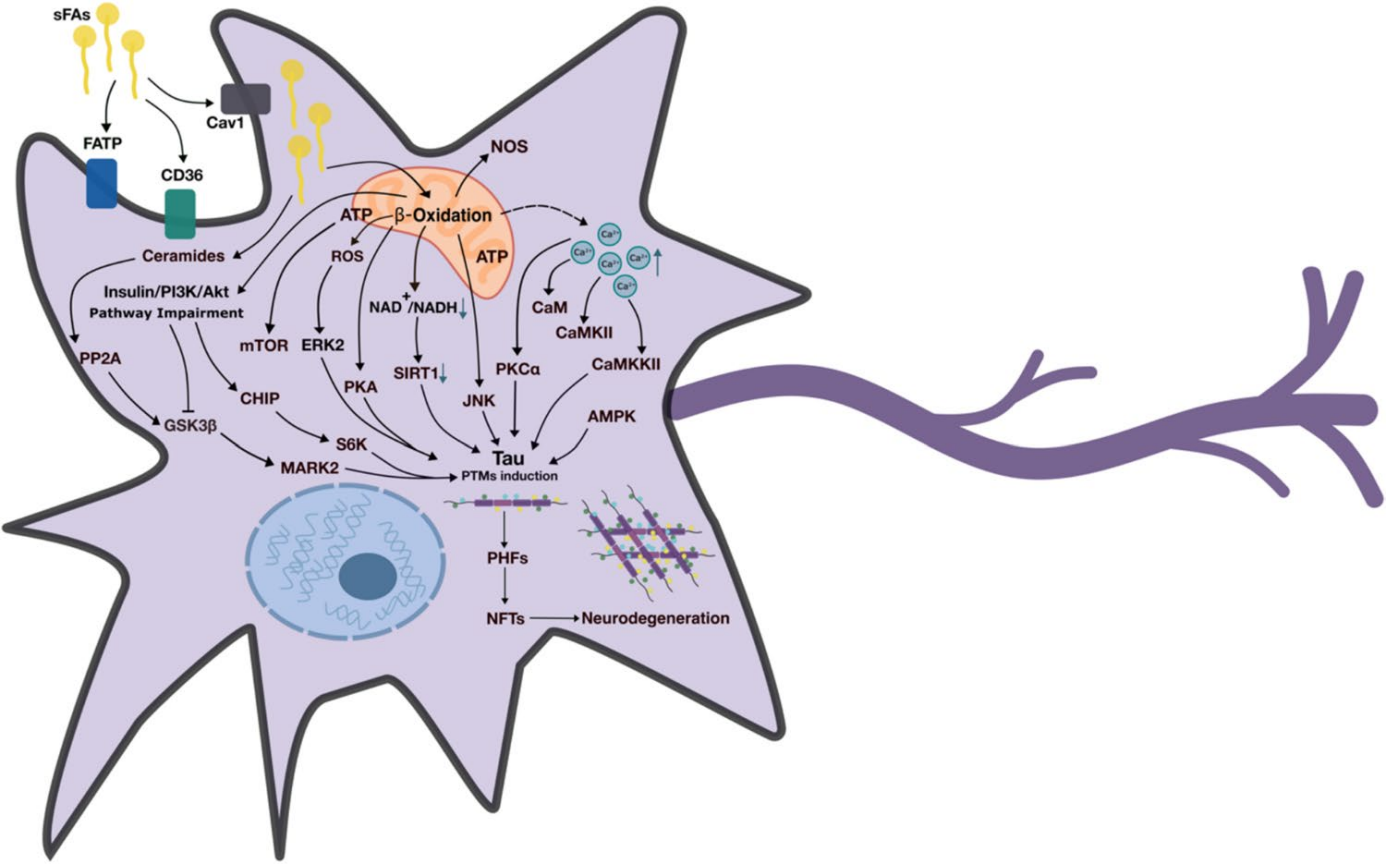
Effects of sFA metabolism on signaling pathways. sFAs metabolism results in the activation of multiple signaling pathways that potentially induce post-translational modifications of Tau leading to destabilization and aggregation into insoluble forms

All the above evidence strongly indicates that the neuronal metabolism of PA contributes to the activation or inactivation of signaling pathways that alter the function of several protein kinases involved in Tau PTMs. In addition to protein kinase modulation, protein acetylation is also increased when a constant high concentration of acetyl-CoA is produced as a result of PA β-oxidation [144]. In hippocampal neurons exposed to high levels of PA, increased acetylation of the transcription factor NF$k$B and reduced levels of Sirt1 were detected [68], and in differentiated human neuroblastoma, hyperacetylation of the Tau protein at the K280 residue was reported [16]. The NAD^+^-dependent deacetylase Sirtuin1 (Sirt1) is one of the main enzymes involved in the removal of the acetyl group from Tau [140]. PA metabolism significantly reduces NAD^+^ levels by targeting nicotinamide phosphoribosyl transferase (NAMPT), the rate-limiting enzyme for NAD^+^ synthesis in hepatic cell lines and primary human hepatocytes [145]. Similarly, a reduction in the NAD^+^/NADH ratio occurs in PA-exposed rat hippocampal neurons associated with Sirt1 deficiency [68]. Deletion of Sirt1 increases the levels of acetylated Tau and pathogenic forms of p-Tau, likely by blocking proteasome-mediated degradation [25]. Acetylation can produce insoluble forms of Tau that also promote its aggregation [42]. Interestingly, the acetylation of different isoforms of Tau differentially impacts its potential for aggregation and fibrillization. For example, when the isoform that contains three repeats (3R) is acetylated at the five lysine residues in the repeated region, fibrillization is accelerated, whereas the isoform with four repeats (R4) is more resistant to aggregation after being acetylated [146]. Figure 2 summarizes the metabolic pathways affected by neuronal exposure to sFA and the enzymes involved in Tau biochemical changes.

## Conclusions and future directions

High consumption of sFAs has been associated with both morphological and functional changes in neurons. Accumulating evidence convincingly shows that PA-induced dysregulation of neuronal metabolism leads to insulin resistance, decreased glycolysis, altered mitochondrial function, and ER stress. These effects appear to contribute to cognitive decline. Recent compelling evidence supports the hypothesis that neurons exposed to high concentrations of saturated long-chain FAs may metabolize them through a metabolic energy pathway that underlies part of their deleterious effects. Although additional experimental data are needed to obtain conclusive results regarding the involvement of sFA-induced metabolic stress in several PTMs of Tau, particularly in in vivo models, the aforementioned evidences strongly suggests the existence of such a link.

In this work, we reviewed the various consequences of PA metabolism in neurons and highlighted the possible relationships between them and the induction of different Tau PTMs, which may lead to a favorable environment for the generation of insoluble forms of Tau. The presented evidence offers valuable insights that could help to explain the link between metabolic disorders associated with lifestyle factors and the increased risk of developing AD.

## Data Availability

No datasets were generated or analysed during the current study.

## Abbreviations

ACOT7: Acyl-CoA thioesterase 7
AD: Alzheimer’s disease
Akt: Protein kinase B
AMP: Adenosine monophosphate
AMPA: α-Amino- 3-hydroxy- 5-methyl- 4-isoxazolepropionic acid receptor
ATP: Adenosine triphosphate
BBB: Blood–brain barrier
CaM: Calmodulin
CAMK2: Calcium/calmodulin-dependent protein kinase II
CDK5: Cyclin dependent kinase 5
CHIP: E3 ubiquitin-protein ligase
CK1: Casein kinase 1
CK2: Casein kinase 2
CPT: Carnitine palmitoyltransferase
ER: Endoplasmic reticulum
ERK1/2: Extracellular signal-regulated kinases
FATP: Fatty acid transporter proteins
Fyn: Proto-oncogene tyrosine-protein kinase Fyn
GPR40: G-protein coupled receptor 40
GSK3β: Glycogen synthase kinase 3 beta
HFD: High-fat diet
Hsc-70: Heat shock cognate 71-kDa protein
JNK: C-Jun N-terminal kinases
MAPKs: Mitogen-activated protein kinases
MARK4: Microtubule affinity-regulating kinase 4
mTOR: Mammalian target of rapamycin
NAD+: Nicotinamide adenine dinucleotide
NAMPT: Nicotinamide phosphoribosyl transferase
NF $k$B: Nuclear factor kappa-light-chain-enhancer of activated B cells
NFT: Neurofibrillary tangles
PA: Palmitic acid
PI3K: Phosphatidylinositol 3-kinase
PKA: Protein kinase A
PKC: Protein kinase C
PP2A: Protein phosphatase 2A
PTMs: Post-translational modifications
ROS: Reactive oxygen species
S6K: Ribosomal protein S6 kinase beta- 1
sFA: Saturated fatty acids
Sirt1: Sirtuin 1
Src: Proto-oncogene tyrosine-protein kinase Src
Ub: Ubiquitin
